# LncRNA MALAT1 acts as a miR-125a-3p sponge to regulate FOXM1 expression and promote hepatocellular carcinoma progression

**DOI:** 10.7150/jca.29213

**Published:** 2019-10-22

**Authors:** Shihai Liu, Jing Qiu, Guifang He, Ye Liang, Liping Wang, Changchang Liu, Huazheng Pan

**Affiliations:** 1 Medical Animal Lab, the Affiliated Hospital of Qingdao University, Qingdao, 266500, China; 2 Department of stomatology, Qingdao Municipal Hospital, Qingdao, 266071, China; 3 Department of Urology, the Affiliated Hospital of Qingdao University, Qingdao, 266500, China; 4 Clinical Lab, the Affiliated Hospital of Qingdao University, Qingdao, 266003, China; 5 Department of Microbiology, Tumor and Cell Biology, Karolinska Institute, Stockholm, 171779, Sweden

**Keywords:** HCC, lncRNA MALAT1, miR-125a-3p, FOXM1

## Abstract

**Background:** Hepatocellular carcinoma (HCC) is a prominent cancer type, with long non-coding RNAs (lncRNAs) being known to be relevant to its progression. We therefore investigated how a particular lncRNA known as the metastasis-associated lung adenocarcinoma transcript 1 (MALAT1) was associated with HCC.

**Methods:** Quantitative reverse transcriptase PCR (qPCR) was used to assess expression of MALAT1, Forkhead Box M1 (FOXM1) and miR-125a-3p in HCC tissue samples. How MALAT1 regulates HCC proliferation and metastasis was assessed through appropriate assays. FOXM1 was identified as a miR-125a-3p target using luciferase assays, and how MALAT1/miR-125a-3p alter FOXM1 expression was explored via qPCR and Western blotting.

**Results:** HCC tissues exhibited MALAT1 upregulation. miR-125a-3p targeted FOXM1 and could be regulated by MALAT1. MALAT1 knockdown disrupted proliferation and invasion, whereas miR-125a-3p knockdown partially reversed this phenotype.

**Conclusions:** These results indicate that MALAT1 modulates FOXM1 expression via being a miR-125a-3p sponge, thus promoting HCC progression.

## Introduction

Hepatocellular carcinoma (HCC) is among the most frequent forms of cancer and a primary cause of human mortality 1. While great advances towards combatting HCC have been made, in general the prognosis of this cancer is still poor 2. It is thus vital that the mechanisms underlying HCC pathogenesis be better understood in order to identify valuable markers of disease progression or prognosis, allowing for novel therapies to be developed 3. Many genes are involved in HCC pathology, including both tumor suppressors and oncogenes, but the entirety of the underlying molecular mechanisms remain to be elucidated.

Long non-coding RNAs (lncRNAs) lack the potential to code for any protein and are longer than 200 nucleotides. LncRNAs have been linked to virtually all processes within cells, such as metabolism, growth, proliferation, and apoptosis 4. Altered lncRNA expression can regulate tumorigenesis, by regulating these same pathways and thereby altering metastasis, growth, and invasion of tumor cells 5. Such altered lncRNA expression has also been shown to be clinically relevant to cancer diagnosis and prognosis determination 6. For example, X-inactive specific transcript (XIST) is a lncRNA that modulates phosphatase and tensin homolog (PTEN) expression and HCC progression 7. Similarly, androgen receptor regulated long noncoding RNA 1 (ARLNC1) is linked with prostate cancer progression 8.

LncRNAs are key mediators of tumorigenesis 9. Roles played by these molecules include acting as guides or decoys that alter and regulate gene expression or even of other non-coding RNA molecules 10. One role of these lncRNAs is as competing endogenous RNAs (ceRNAs), while they can also be molecular “sponges” that thereby alter miRNA expression or function 11. HOXD cluster antisense RNA 1 (HOXD-AS1), as an example, drives the metastasis of HCC via performing a ceRNA function, leading to SRY-related HMG-box 4 (SOX4) upregulation and activation of SOX4 target genes 12. In contrast, cancer susceptibility candidate 2 (CASC2) is a lncRNA with tumor suppressor functionality, acting as a ceRNA for miR-367 and thereby reducing changes in invasive potential/Epithelial-to-mesenchymal transition (EMT) for HCC cells 13.

Metastasis-associated lung adenocarcinoma transcript 1 (MALAT1) is a lncRNA linked with the breast, lung, and other cancer development 14. MALAT1 has also been shown to confer resistance to Docetaxel (DTX) resistance via A-kinase anchoring protein 12 (AKAP12) in prostate cancer (Pca) cells 15. We were therefore interested in confirming whether MALAT1 was involved in the regulation of HCC, and if so by what mechanisms it functioned in this context. In particular, the starBase v2.0 software platform suggested that MALAT1 be a miR-125a-3p sponge, regulating its expression. FOXM1 is known to be another gene relevant to the progression of HCC and other forms of cancer 16. miR-125a-3p was predicted to bind to the 3'UTR of FOXM1, leading us to hypothesize that MALAT1 could target miR-125a-3p and thereby regulate FOXM1, thus altering HCC oncogenesis. Consistent with our hypothesis, we found elevated MALAT1 levels in HCC samples and cells that correlated with more advanced cancers. By knocking down MALAT1, we disrupted HCC cell proliferation and invasive potential, and we were further able to confirm the role of this lncRNA as a miR-125a-3p sponge. These results ultimately confirmed that MALAT1 can contribute to HCC oncogenesis through regulation of FOXM1 by way of miR-125a-3p. These novel results shine new light on how lncRNAs can indirectly and directly modulate HCC progression and development.

## Materials and Methods

### Patient samples and cells

All patients provided written and informed consent for this study. A total of 30 HCC and matched normal tissue samples were collected following hepatectomy in HCC patients from the Affiliated Hospital of Qingdao University, China, between January 2010 and December 2011. None of the patients had received any adjunctive therapy. Inclusion criteria were as follows: 1) patients had not received radiofrequency ablation preoperatively; 2) Child-Pugh A/B; and 3) no concurrent cancers. Exclusion criteria were as follows: 1) recurrent HCC; 2) surgery-related death within 10 days after surgery; 3) extrahepatic metastasis; and 4) incomplete follow-up data. Huh-6, HuH-7, PLC, HCCLM3, SK-HEP1, and L02 cell lines were all purchase from Shanghai Institutes for Biological Sciences of Chinese Academy of Sciences, China. Culture was conducted in a 37 °C 5 % CO_2_ incubator using RPMI 1640 supplemented with 10% FBS and appropriate antibiotics.

### RNA exaction, reverse transcription and quantitative real-time PCR (qPCR)

Trizol (Takara, Shiga, Japan) was used for all RNA extractions, and DNase I (Takara) was used to eliminate DNA from these samples. cDNA was synthesized from 1 μg RNA with a Reverse-Transcribe Kit (Takara). qPCR was used for miR-125a-3p detection by combining miRNA-specific primers with RNA and M-MLV RT (Takara). U6 served as the reference control. To assess expression of MALAT1 and FOXM1 mRNA, the reference control was GAPDH. Primer sequences were: FOXM1-F, 5'- ATACGTGGATTGAGGACCACT-3', FOXM1-R, 5'- TCCAATGTCAAGTAGCGGTTG-3'; MALAT1-F, 5'- ATCTGCAAAACAAAAA CCCCT -3'; MALAT1-R, 5'-GTCTCCGAAGACACAGAGACCT-3'; GADPH-F, 5'-ACTGCCACCCAGA AGACT-3', GADPH-R, 5'-GCTCAGTGTA GCCCAGGAT-3'. qPCR was conducted in triplicate using a LightCycler 480 (Roche, Switzerland). All the real-time PCR primers were derived from the published work 17, 18. The PCR reaction system for miRNA was as follows: initial denaturation at 95 °C for 20 s and 30 cycles of 95 °C for 10 s, 60 °C for 20 s, and 72 °C for 10 s. For lncRNA, the reaction system was as follows: initial denaturation at 95 °C for 30 s and 35 cycles of 95 °C for 5 s and 60 °C for 20 s. The threshold cycle was determined after the reactions. The relative miRNA and lncRNA expression levels were calculated based on comparative threshold cycle (Ct) technique (2^-(ΔΔCt)^) . The expression level was normalized to the U6 or GAPDH levels of each sample, respectively.

### Cell transfection

Mimics, inhibitors, and control samples for miR-125a-3p were synthesized to order (GenePharma, China). siMALAT1 and control (siR-NC) siRNAs were from the same source (GenePharma). Lipofectamine 2000 (Invitrogen) was utilized for all transfections based on manufacturer's protocols.

### Cell proliferation assay

HCC cells (5 × 10^4^) were added in 24-well plates followed by transfection of indicated RNA constructs or controls. After 1, 2, and 3 days absorbance was measured at 450 nm via Multiskan GO (Thermo Fisher Scientific, USA).

When EdU uptake was being assessed 24 hours after transfection with appropriate mimics, inhibitors, or control constructs, HCC cells were labeled with EdU and imaged using the BeyoClick™ EdU-555 Imaging Kit (Beyotime, Beijing, China). DAPI was used to stain nuclei in this experiment, and the number of EdU+ cells was determined as a readout for proliferation.

### Wound-healing assay

HCC cells (5 × 10^6^) were added per well of a 6 well plate, following transfection and 24 hours of serum starvation. Media was then replaced, and a scrape was made to simulate wounding. Twenty-four hours later, the closing of this wound was imaged using a microscope (×40) in triplicate.

### Transwell assays

Eight μm polycarbonate transwell filters (Corning Inc., Corning, NY) were put in 20% fetal bovine serum (FBS) media containing wells, and 4 × 10^4^ cells in 1% FBS media were seeded in the upper well followed by incubation at 37°C for 24 hours. Cotton swabs then removed cells failing to migrate, while 4 % paraformaldehyde was used to fix migratory cells, which were stained with 0.4% crystal violet. A microscope was used to photograph these cells, and five random fields were enumerated. The invasion assay was performed in the same manner, with upper chambers containing 1 mg/mL Matrigel (Corning Inc.).

### Western blotting analysis

Western blotting was based on previous protocols 19. The 1 × SDS buffer was used for lysate preparation, with the protein quantification via BCA Protein Assay (Thermo Fisher Scientific). Thirty µg total protein was run on an Sodium Dodecyl Sulfate - Polyacrylamide Gel Elecrtophoresis (SDS-PAGE) gel and transferred to nitrocellulose membranes. Membranes were incubated in solutions with antibodies against FOXM1 (1:500 dilution, Cell Signaling Technology #5436), E-cadherin (1:1000 dilution, Cell Signaling Technology #3195), N-cadherin (1:500 dilution, Cell Signaling Technology, #13116) or glyceraldehyde-3-phosphate dehydrogenase (GAPDH) (1:2000 dilution, Cell Signaling Technology, #5174) overnight at 4 °C. A secondary anti-Ig HRP-linked Ab was then incubated on blots, followed by detection via enhanced chemiluminescence (ChemiDoc XRS+, Bio-Rad, Hercules, CA, USA). GAPDH served as a loading control.

### Luciferase reporter assays

Plasmids for luciferase reports that contained the wild-type (WT) or mutant (MUT) MALAT1 were created internally, with phRL-TK as an internal control (Promega, Madison, WI). HCC cells (2 × 10^6^) in 24 well plates received these plasmids along with miR-125a-3p mimics, controls, or miR-125a-3p inhibitors or miR-NC as appropriate for a total of 24 hours. Cells were washed and lysed via passive lysis buffer (Promega). A 96-well plate luminometer (Multiskan GO, Thermo Fisher Scientific) was then used to analyze these lysates, and luciferase activities were quantified with Dual-Luciferase Reporter Assay System kit (Promega) based on provided protocols.

A pGL6-miR vector was created via insertion of the FOXM1 3'-UTR containing the miR-125a-3p binding site: h-FOXM1-3UTR-F: 5' - CGGGATCCAGCAGTCACACCCTAGCCACTG -3' and h-FOXM1-3UTR-R: 5' - CCGCTCGAGAGTTAGTGTTCTCAAGCTGGC -3' using the BamHI and XhoI sites. A control vector - the 3'-UTR fragment containing binding site mutations was generated: h-FOXM1-mut-F: 5' - CCTCCCCGCAACACGCTTACGCTATGCATCCATAGA -3', h-FOXM1-mut-R: 5' - ATTTCTTCTATGGATGCATAGCGTAAGCGTGTTGCGG - 3'. HCC cells were with these plasmids in reporter assays, along with the miR-125a-3p mimic or control. Luciferase activities were assessed 24 hours later with the Dual-Glo™ Luciferase Assay System as above. Renilla luciferase activity served as a control, with the Firefly/Renilla activity being used for normalization.

### Tumor xenografts in nude mice

Four to six week old female athymic nude mice (Beijing Vital River Laboratory Animal Technology Co., Ltd.), were housed at the Affiliated Hospital of Qingdao University. Animal studies were conducted in accordance with the Affiliated Hospital of Qingdao University Institutional Animal Care and Use Committee guidelines. All survival studies utilized humane end points. To collect livers, mice were euthanized via CO_2_ asphyxiation. Mice received subcutaneous injections of, for Group A (n = 6), HCCLM3 cells infected with control lentiviruses (LV-siCon), or for group B (n = 6), HCCLM3 cells infected with shMALAT1 (LV-shMALAT1). Tumor growth was monitored every two days using calipers for 24 days total, after which animals were sacrificed by CO_2_ asphyxiation. Resected samples were used for immunohistochemical analysis.

### Hematoxylin and Eosin (H&E) Staining

Buffered formalin (10%) was used to fix liver samples. Sections with 5 μm thick were placed on slides, deparaffinized, and stained with hematoxylin and eosin. Changes in morphology were visualized using a light microscope.

### Statistical analyses

SPSS 16.0 was used for all statistical testing. MALAT1, FOXM1 or miR-125a-3p expression comparisons relied upon the Student's t-test. Correlation assessments were performed via Pearson's analysis. The Kaplan-Meier method was used for survival curves. All data are means ± SD. The significance threshold was *P* < 0.05.

## Results

### HCC tissues have elevated MALAT1 levels that correlated with cancer progression

**Figure 1:**
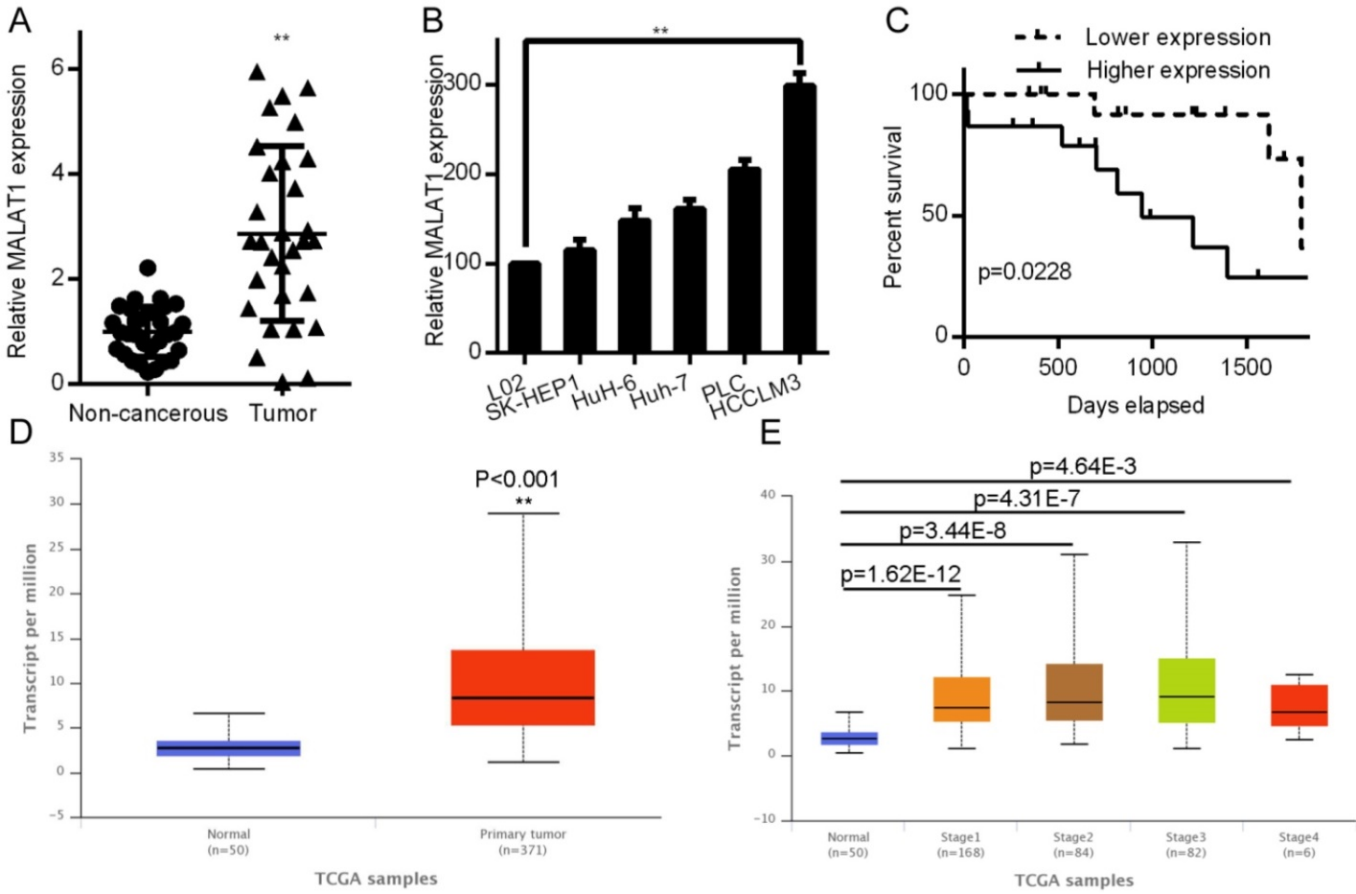
MALAT1 upregulation was linked poor HCC outcomes. A. Relative HCC tissue MALAT1 levels relative to non-cancerous samples as assessed by qPCR. B. MALAT1 levels in HCC cell lines were quantified by qPCR. C. A Kaplan-Meier curve was used to compare survival in high and low MALAT1 expressing patients, *P* < 0.05. D. LIHC and normal control samples in the TCGA dataset were compared for MALAT1 expression, using 371 LIHC and 50 controls samples from UALCAN: http://ualcan.path.uab.edu/index.html. E. MALAT1 mRNA in LIHC samples for each stage was assessed in TCGA samples.

MALAT1 has been shown to be altered in a relevant fashion in lung, breast, prostate, and gallbladder cancers 20-22. We therefore assessed MALAT1 expression in samples from paired HCC and non-cancerous samples (n = 30) by qPCR. MALAT1 levels were significantly elevated in HCC samples (*P*< 0.001; Fig. 1A). We also analyzed HCC cell line expression of MALAT1, where it was also upregulated relative to the control L02 cell line (Fig. 1B). A Kaplan-Meier analysis was employed to investigate how MALAT1 expression associated with overall patient survival. Those patients with higher MALAT1 expression were found to have overall poorer survival (*P* < 0.05, Fig. 1C). This was consistent with MALAT1 expression levels in 371 LIHC patients compared to 50 control patients in the Cancer Genome Atlas (TCGA) dataset (http://ulcan.path.uab.edu/index.html, 23), where MALAT1 levels were higher in Liver hepatocellular carcinoma (LIHC) patients (Fig. 1D). Expression of MALAT1 was higher for all LIHC stages relative to controls (Fig 1E). Together, these results indicate a clear link between MALAT1 expression and HCC development or progression.

### MALAT1 drives HCC proliferation, invasion, migration, and epithelial mesenchymal transition (EMT)

**Figure 2:**
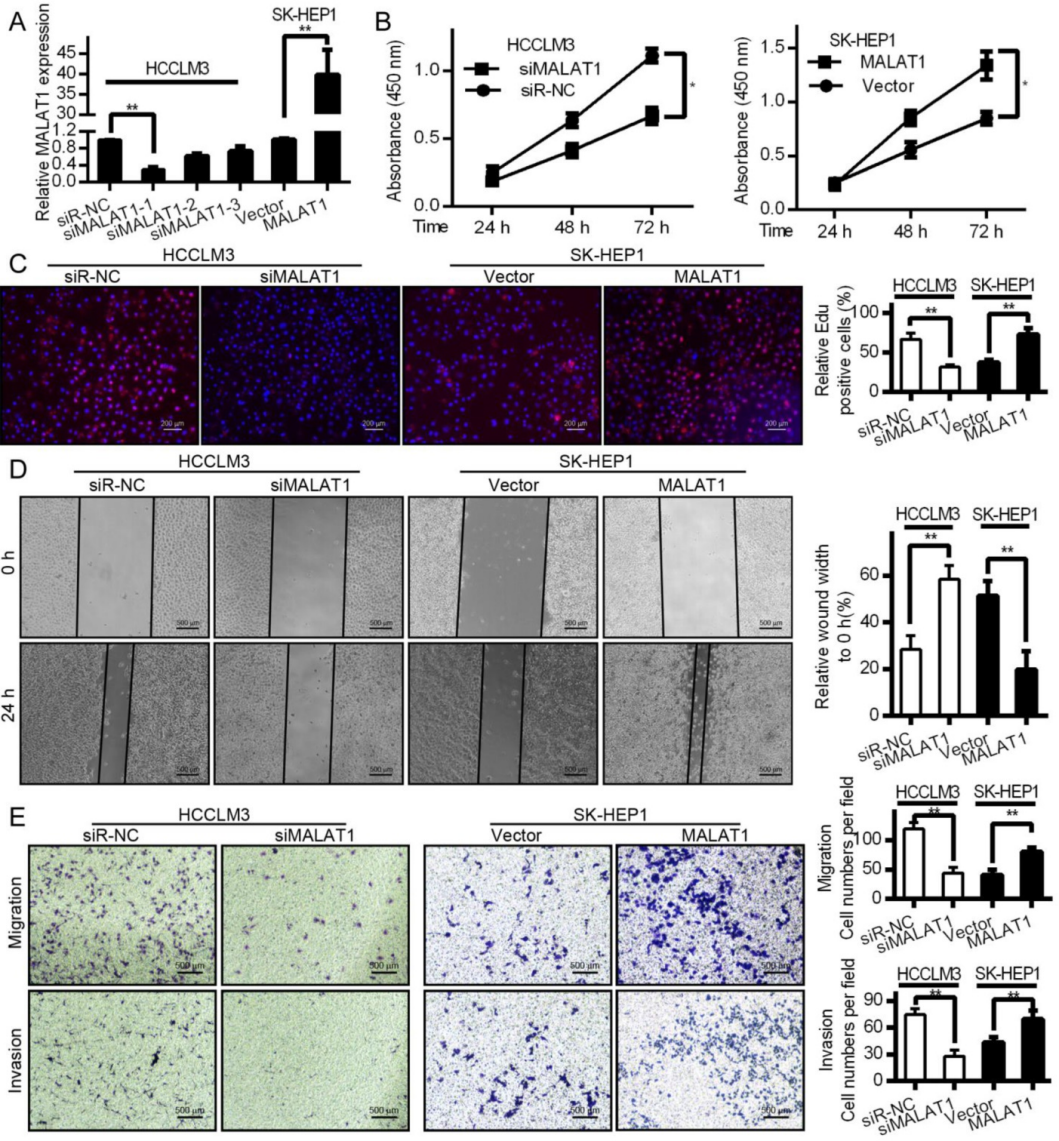
MALAT1 regulates in vitro HCC proliferation, migration and invasion. A. siMALAT1 dramatically reduced expression of MALAT1 relative to control (siR-NC) in HCCLM3 cells. MALAT1 overexpression was performed in SK-HEP1 cells. B. CCK-8 proliferation showed that MALAT1 knockdown decreased proliferation of HCC cells, while its overexpression increased it. C. EdU assays indicated that modulating MALAT1 altered HCC cell proliferation. D. Cell line wound healing assays for cell lines with altered MALAT1 expression. D. MALAT1 knockdown impaired the migration of tumor cells in wound healing assays. E. Transwell migration and matrigel invasion assays indicating that MALAT1 knockdown and overexpression modulates these parameters. Results are means ± SD for experiments in triplicate. *P<0.05; ** *P*<0.01.

Both proliferation and invasion are important indicators of cancer 24. To test how they are influenced by MALAT1, we knocked down its expression with siRNA in HCCLM3 cells and overexpressed it in SK-HEP1 cells. qPCR was used to measure transfection efficiencies (Fig. 2A). CCK-8 proliferation assays shown that MALAT1 promoted HCC cell viability (Fig. 2B). EdU assays also showed a role for MALAT1 in promoting HCC proliferation (Fig. 2C), confirming the importance of MALAT1 for HCC proliferation. MALAT1 downregulation significantly slowed cell migration in a wound healing assay, while overexpression enhanced migration (Fig. 2D). This was also confirmed by Transwell assay. MALAT1 knockdown for 24 h led to reduced HCCLM3 migration (Fig. 2E), while its over-expression enhanced SK-HEP1 cell migration (Fig. 2E). Invasive potential also decreased upon MALAT1 knockdown as determined with a Matrigel Transwell assay, with the opposite result upon MALAT1 upregulation (Fig. 2E). This clearly shows a role for MALAT1 in HCC proliferation, migration, and invasion.

**Figure 3:**
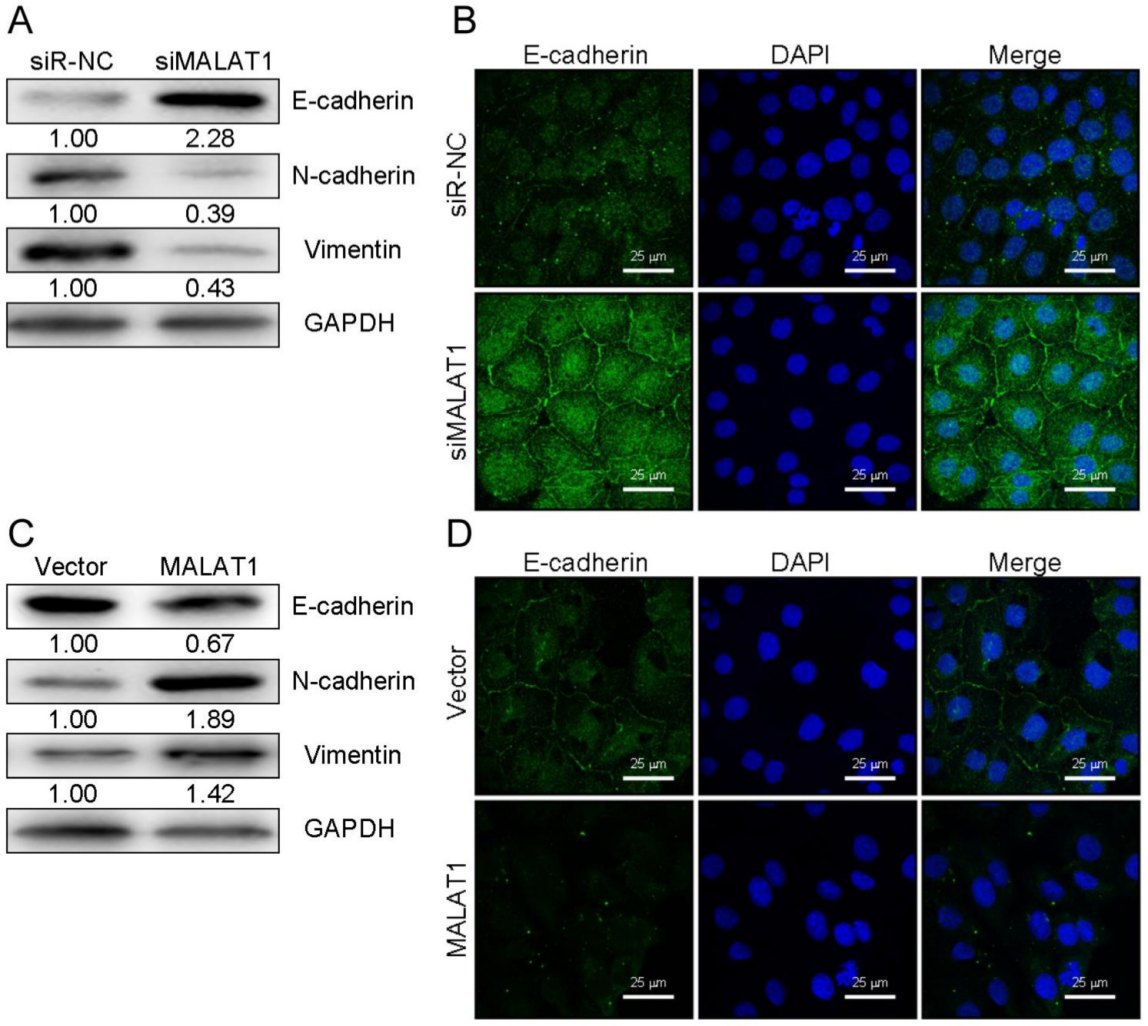
MALAT1 expression modulates EMT markers in HCC cells. (A and C) E-cadherin, N-cadherin, SNAIL, and Vimentin levels were measured by Western blotting after knockdown (HCCLM3) or overexpression (SK-HEP1) of MALAT1, with GAPDH as a loading control. (B and D) E-cadherin levels were assessed by immunofluorescence following MALAT1 knockdown (HCCLM3) or overexpression (SK-HEP1). Scale bars are 25 µm.

EMT is a key metastatic step in cancer progression 25. We next wanted to examine changes in EMT markers associated with overexpression or knockdown of MALAT1 in HCC cells. Western blotting revealed that knocking down MALAT1 in HCCLM3 cells led to elevated E-cadherin expression (an epithelial marker) and decreased expression of SNAIL, Vimentin, and N-cadherin. Overexpression yielded the opposite phenotype for these markers (Fig. 3A and 3C). Comparable results were obtained with regard to E-cadherin levels in SK-HEP1 cells as measured by immunofluorescence (Fig. 3B and 3D). This suggests a role for MALAT1 in HCC cell progression via EMT regulation, though additional work is needed to investigate the underlying mechanisms.

### MALAT1 regulates FOXM1 by informatics analysis and identification

**Figure 4:**
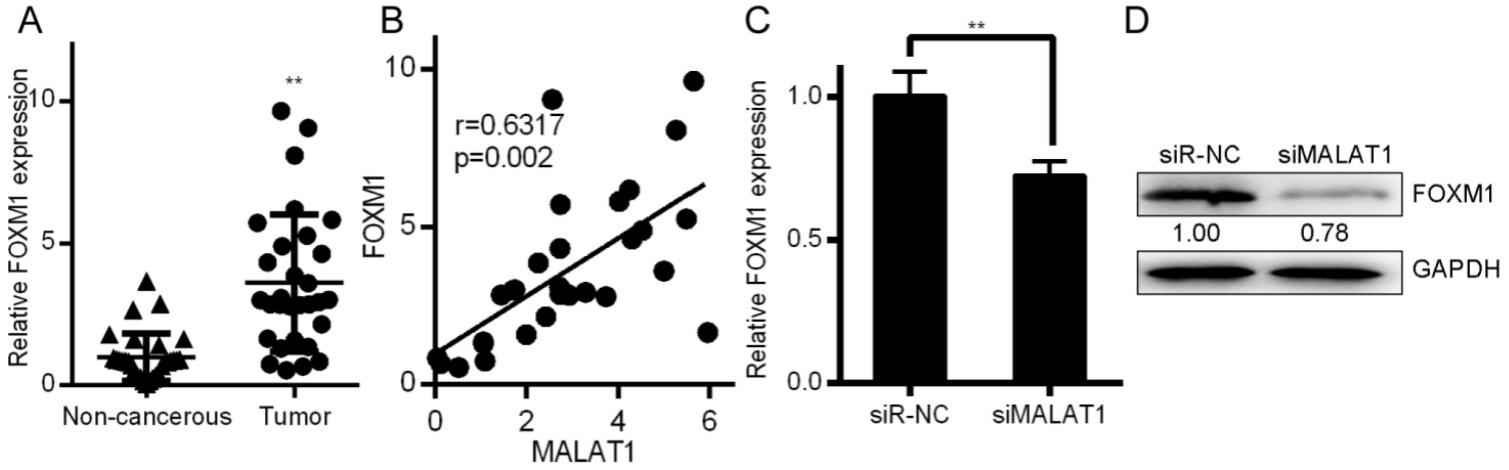
MALAT1 regulates FOXM1 expression. A. Relative FOXM1 expression in HCC tissues (n=30) relative to normal controls (n=30), as determined by qPCR with GAPDH normalization. B. A MALAT1 and FOXM1 correlation analysis was conducted for these HCC patients. C. FOXM1 levels in HCCLM3 cell lines transfected with control siRNA (siR-NC) and siMALAT1 were measured. D. Protein levels of FOXM1 were measured in these same HCCLM3 cell lines. Data are means ± SD of 3 samples. **P<0.01.

Associations between the MALAT1 lncRNA and expression of other mRNAs were assessed using the LinkedOmics database (http://www.linkedomics.org/), which has data regarding HCC in 371 patients incorporated into the TCGA project 26. This database allowed us to perform Pearson's correlations to compare expression of MALAT1 and other mRNAs (Supplementary Figure 1A and 1B). Among those identified, MALAT1 was associated with FOXM1 expression at p=0.0119 (Supplementary Figure 1C). This was consistent with findings in the Starbase database (Supplementary Figure 1D). Metabolic gEne RApid Visualizer (MERAV) (http://merav.wi.mit.edu/) was used to assess FOXM1 expression in HCC tissues, where it was found to be upregulated (Supplementary Figure 1E). A Kaplan-Meier curve also showed that FOXM1 was predictive of patient survival for these HCC patients (Supplementary Figure 1F). FOXM1 is a transcription factor is involved in tumor progression via EMT regulation, much as we found MALAT1 to regulate this process 27. FOXM1 was detected at higher levels in HCC tissues relative to controls (Fig. 4a). MALAT1 was also positively correlated with FOXM1expression (Fig. 4B). FOXM1 protein and mRNA was also downregulated in HCCLM3 cells upon siMALAT1 treatment (Fig. 4C and 4D), suggesting that MALAT1 upregulates FOXM1 in HCC cell lines.

### MALAT1 binds and blocks expression of miR-125a-3p

**Figure 5:**
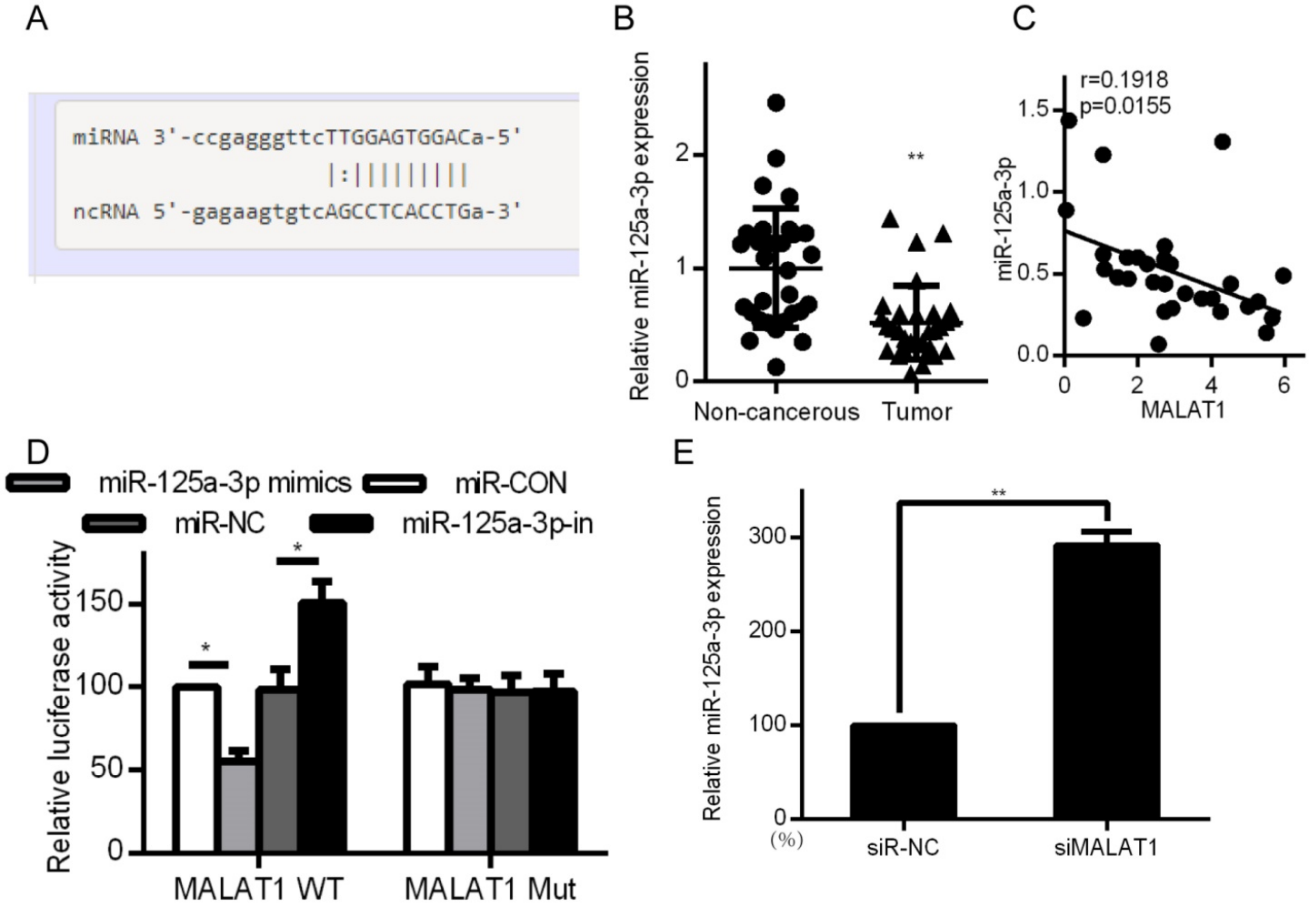
MALAT1 binds to and suppresses miR-125a-3p expression. A. Putative miR-125a-3p binding sites in MALAT1. B. miR-125a-3p expression in HCC tissue samples (n=30) relative to normal tissues (n=30), measured by qPCR. C. Correlation between MALAT1 and miR-125a-3p expression in HCC patients. D. Readouts following WT/MUT MALAT1 luciferase reporter transfection in HCCLM3 cells that also received miR-125a-3p mimics or controls. E. miR-125a-3p expression in HCCLM3 cell lines in which MALAT1 was knocked down. All values are as means ± SD. ** *P* < 0.01.

Certain lncRNAs are known to serve as ceRNAs in the context of cancer, acting as sponges to alter miRNA activity, effectively shielding mRNAs from miRNA targeting 28, 29. We therefore wanted to know whether similar mechanisms were relevant in the context of MALAT1 and its contributions to cancer development. Starbase 2.0 was therefore employed to identify 27 possible miRNAs that were predicted to interact with MALAT1 (Supplementary Table 1). We measured the expression of those miRNAs in siMALAT1 or siR-NC HCCLM3 cells (Supplementary Table 2). The most highly upregulated miRNA in cells in which MALAT1 had been knocked down was miR-125a-3p. We also identified the presence of a miR-125a-3p binding site in MALAT1 (Fig. 5A). Upon analyzing HCC tissue samples, we found substantially decreased miR-125a-3p levels relative to normal tissue controls (Fig. 5B). We also identified a negative correlation between miR-125a-3p and MALAT1 expression (Fig. 5C). We then co-transfected HCCLM3 cells with reporter plasmids for WT or mutated MALAT1 as well as miR-125a-3p mimics or control. WT MALAT1 and miR-125a-3p mimic transfection led to a substantial drop in luciferase activity, consistent with miR-125a-3p binding to MALAT1 (Fig. 5D). qPCR also confirmed elevated miR-125a-3p levels in HCC cells after MALAT1 knockdown (Fig. 5E). Together this shows that miR-125a-3p binds directly to MALAT1, leading MALAT1 to suppress expression of this miRNA.

### FOXM1 is a miR-125a-3p target

**Figure 6:**
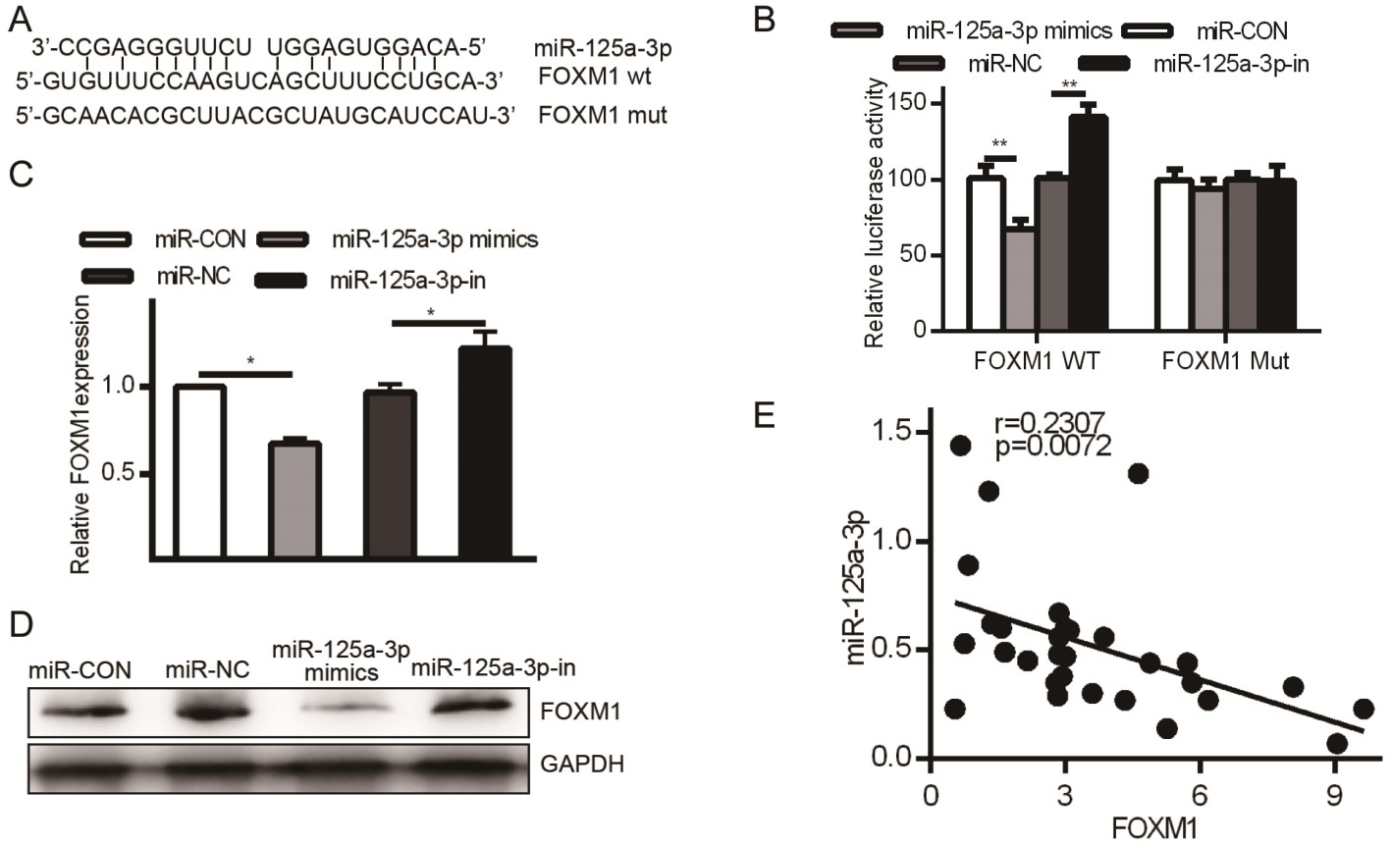
FOXM1 is a miR-125a-3p target. A. Sites of interaction between the FOXM1 3' UTR and miR-125a-3p. B. Reporter assay results following WT/MUT FOXM1 and miR-125a-3p mimic transfection. C. FOXM1 mRNA expression in HCC cells following miR-125a-3p mimic transfection. D. Western blotting testing total FOXM1 protein in HCC cells after miR-125a-3p transfection. Data are shown as means ± SD of 3 samples. **P<0.01; *** *P*<0.001.

HCC patient tissue samples exhibited much higher FOXM1 expression than did matched normal samples, but we had not yet assessed the relationship between FOXM1 and miR-125a-3p. miRanda revealed FOXM1 as a miR-125a-3p target, binding to a region in FOXM1 mRNA 3' UTR, FOXM1 (Fig. 6A). To confirm this prediction, we transfected HCCLM3 cells with either WT or MUT FOXM1 3' UTR luciferase reporter plasmids along with the miR-125a-3p mimics or controls as above, and then assessed luciferase activity. This analysis showed reduced FOXM1 translation in the presence of miR-125a-3p mimic transfection, while MUT-FOXM1 translation was not altered in this context (Fig. 6B). FOXM1 mRNA and protein levels were also measured after miR-125a-3p mimics or control transfection. This revealed significantly reduced FOXM1 levels upon miR-125a-3p mimic transfection relative to control transfection (*P* < 0.01).

### FOXM1 levels were altered by MALAT1 knockdown via miR-125a-3p, influencing invasive activity

**Figure 7:**
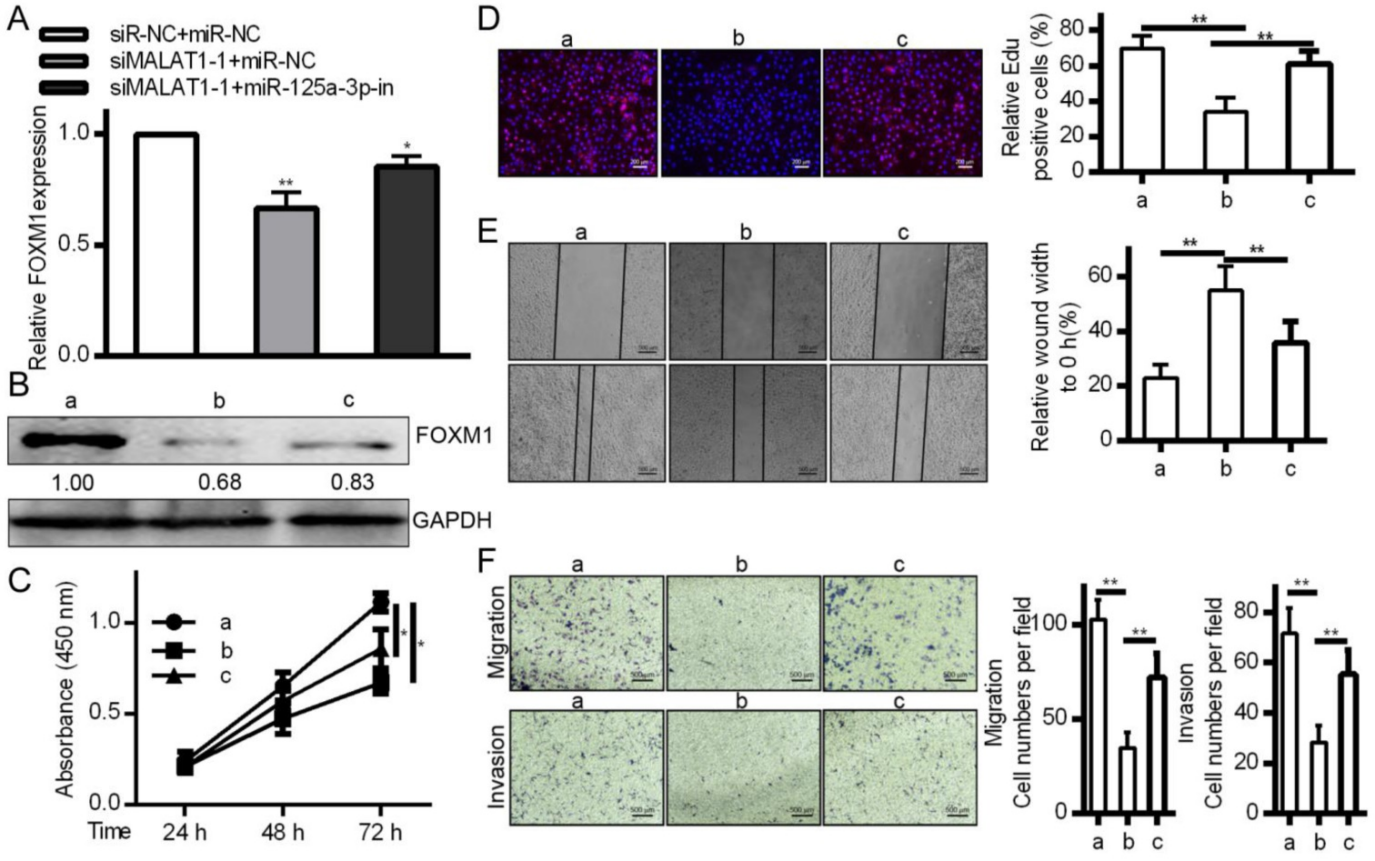
MALAT1 inhibition regulates the expression of FOXM1 HCC cell proliferation, migration and invasion by miR-125a-3p. A. FOXM1 mRNA levels showing decreased expression in response to MALAT1 knockdown, reversed via miR-125a-3p inhibition. B. Western blotting of FOXM1 protein levels indicating that miR-125a-3p inhibition reverses decreased FOXM1 levels observed upon MALAT1 knockdown. C. CCK-8 confirmed that inhibiting miR-125a-3p reversed the changes in cell proliferation identified upon MALAT1 knockdown. D. Wound healing assays demonstrating how inhibition of miR-125a-3p reversed changes in HCC cell migration upon MALAT1 knockdown. E. Transwell migration assays demonstrating that miR-125a-3p inhibition reversed changes in migration that arose upon MALAT1 knockdown. F. Matrigel chamber invasion assays demonstrating that miR-125a-3p inhibition reversed changes in migration upon MALAT1 knockdown. Data are shown as means ± SD of 3 samples. *P<0.05; **P<0.01.

To affirm the link between MALAT1, miR-125a-3p, and cancer progression, MALAT1 was knocked down with or without a miR-125a-3p inhibitor. This led to reduced FOXM1 levels in response to MALAT1 knockdown, and this was reversed by miR-125a-3p inhibition (Fig. 7A and 7B), suggesting inhibition of miR-125a-3p may be how MALAT1 influences FOXM1 expression. We then tested whether miR-125a-3p knockdown reversed the effects of MALAT1 knockdown in these same cells, and we determined that miR-125a-3p inhibition could partially reverse defects in proliferation, migration, and invasion identified in response to MALAT1 knockdown (Fig. 7C, 7D, 7E and 7F). MALAT1 thus acts as a suppressor of miR-125a-3p, thereby possessing oncogenic activity. This shows a direct role for MALAT1 in FOXM1 regulation altering HCC cell proliferation, migration and invasion via miR-125a-3p.

### Inhibition of HCC cells tumorigenesis by MALAT1 knockdown

**Figure 8:**
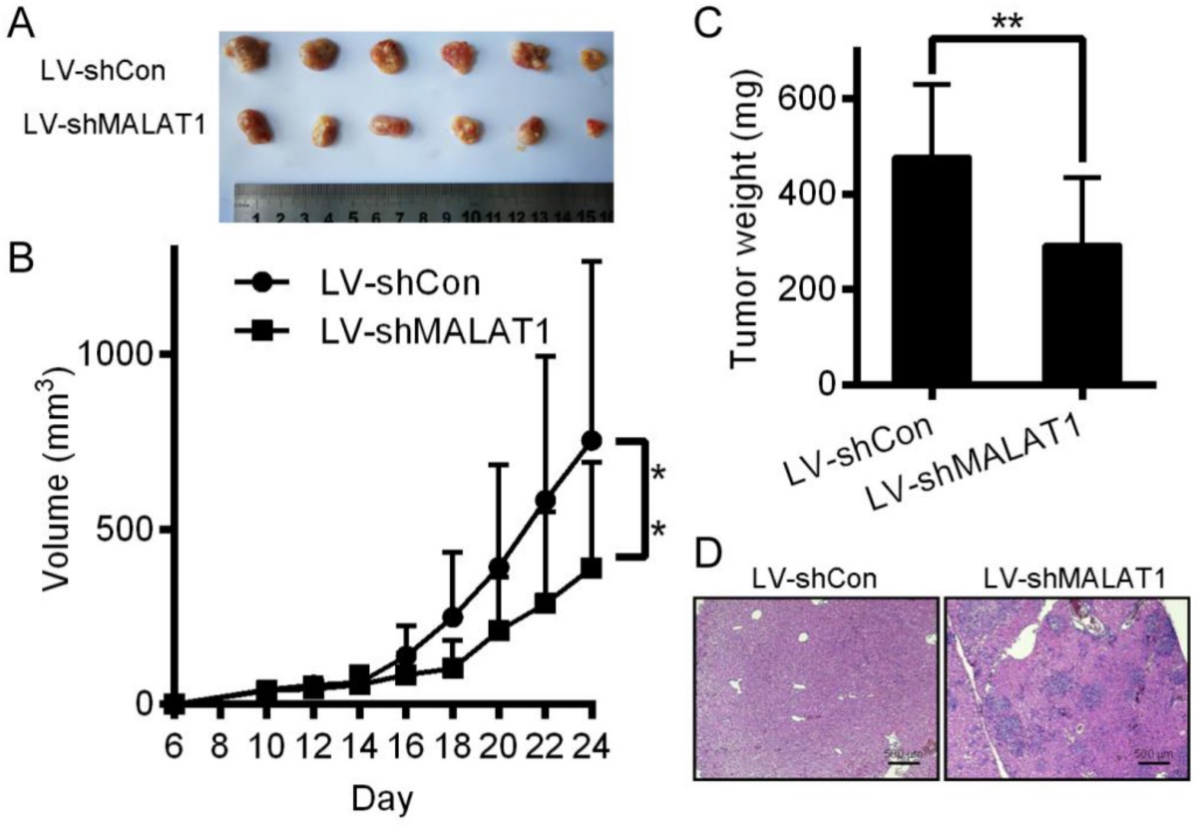
Inhibition of HCC cells tumorigenesis by MALAT-1 knockdown. (A) Tumor images 24 days after LV-shCon or LV-shMALAT1 cell injection in mice. (B) Tumor growth curves for LV-shCon or LV-shMALAT1 HCC cells. (C) Tumor weights, measured after 24 days. (D) Photographs (×40) of H&E staining of the tumors. *P < 0.05, **P < 0.01.

Relative to controls, knocking down MALAT1 in HCC disrupted tumor growth in mice (Fig. 8A, 8B and 8C). H&E staining showed that tumors in the LV-shCon group were more metastatic than those in the LV-shMALAT1 group (Fig. 8D), showing that MALAT1 regulates HCC migration and invasion both *in vivo*.

## Discussion

Herein, we assessed the relevance of the previously described MALAT1 lncRNA in the context of hepatocellular carcinoma, demonstrating its upregulation in HCC tumor tissues relative to normal liver controls. This elevated MALAT1 expression was positively correlated with metastasis and patient clinical stages, and negatively correlated with patient overall survival. By knocking down or overexpressing MALAT1, we identified its role in promoting the migratory and proliferative capacity of HCC cells, highlighting its status as an oncogene in this form of cancer. FOXM1 was also known to have relevance to many forms of cancer, including HCC 16. We were also able to observe FOXM1 upregulation in tissue samples from HCC patients that correlated with MALAT1 levels. MALAT1 inhibition reduced FOXM1 levels at both the mRNA and protein level.

lncRNAs and other nucleic acids have been found to act as ceRNAs 11. Phosphatase and tensin homolog pseudogene (PTENP1) can bind miR-19b and miR-20a in competition with PTEN such that a decrease in PTENP1 levels leads to the depletion of normal PTEN by these miRNAs 30. Similarly, lncRNA-ATB competitively binds miR-200, preventing its binding to zinc finger E-box binding homeobox 1 (ZEB1) and zinc finger E-box binding homeobox 2 (ZEB2) and promoting invasive signaling in HCC cells 31. We similarly showed a role for MALAT1 as a miR-125a-3p sponge, as MALAT1 knockdown increased miR-125a-3p levels in HCC cells. miR-125a-3p levels were also decreased in HCC tissue samples relative to normal controls, and MALAT1 levels were inversely correlated with those of miR-125a-3p.

miRNAs can also regulate cancer progression via direct regulation of specific coding mRNAs. We observed FOXM1 upregulation in the context of HCC tissues, with FOXM1 being a miR-125a-3p target. Given the role for MALAT1 in FOXM1 regulation, we surmised that miR-125a-3p mediated the MALAT1-dependent regulation of FOXM1 levels. This was confirmed by experiments wherein miR-125a-3p inhibition ablated MALAT1 knockdown-inducted changes in FOXM1 levels. miR-125a-3p inhibition also overcame the effects of MALAT1 knockdown on HCC proliferation and migration, suggesting a role for MALAT1 in regulating these processes by means of miR-125a-3p.

In summary, MALAT1 levels are increased in both HCC tissues and cell lines, and this lncRNA has potential as a biomarker of disease progression or prognosis. MALAT1 binds miR-125a-3p, modulating its activity within the cell. FOXM1 is also expressed at higher levels in HCC tissues, serving as a miR-125a-3p target. MALAT1 thereby modulates HCC cell migration and invasion via miR-125a-3p mediated FOXM1 regulation. These results provide important perspective on novel HCC molecular mechanisms, potentially providing new diagnostic insights.

## Supplementary Material

Supplementary figures and tables.
