# Supplementary material for: LncRNA MALAT1 acts as a miR-125a-3p sponge to regulate FOXM1 expression and promote hepatocellular carcinoma progression
Source: J Cancer. 2019 Oct 22;10(26):6649–59. doi: 10.7150/jca.29213 (PMC6856878; doi:10.7150/jca.29213)
Supplement: Supplementary file 1 — Supplementary figures and tables. [file jcav10p6649s1.pdf]

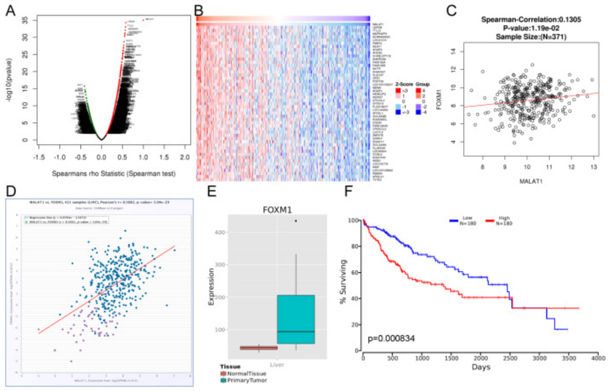

Supplementary Figure 1. Distribution of lncRNA MALAT1 and FOXM1 genes expression between HCC tumor tissue and adjacent normal tissues using the web server. (A and B) Data is acquired from TCGA database and analyzed via LinkedOmics bioinformatics. (C) The relationship between lncRNA MALAT1 and FOXM1 via LinkedOmics bioinformatics. (D) The relationship between lncRNA MALAT1 and FOXM1 via starbase database. (E) Distribution of FOXM1 expression between HCC tumor tissue and adjacent normal tissues using the MERAV web server. (F) FOXM1 gene is related with the overall survival rate of HCC patients. Red line: high expression, blue line: low expression.

E: The expression of FOXM1 in HCC patients. The results, based on TCGA database, were acquired from GEPIA bioinformatics [GEPIA: a web server for cancer and normal gene expression profiling and interactive analyses.
Tang Z, Li C, Kang B, Gao G, Li C, Zhang Z
Nucleic Acids Res. 2017 Jul 3; 45(W1):W98-W102.].

The interactions between miRNAs and mRNAs were provided from starbase.

Kaplan-Meier analysis also indicated that these miRNAs were not suitable for use as prognostic factors for survival outcomes in patients with HCC (Fig. 4), which was also confirmed by LinkedOmics analysis using 453 overlapping samples from the miRNASeq and clinical datasets (hsa-miR-409-3p, P=0.296; hsa-miR-339-5p, P=0.426; hsa-miR-127-3p, P=0.122; has-miR-654, P=0.071).

To find the potential targets of lncRNA MALAT1 that might be involved in HCC carcinogenesis, we searched the candidate targets by using the bioinformatics tools. Interestingly, among all 7 members of sirtuin family, we found that 5 members, including SIRT2, SIRT3, SIRT5, SIRT6 and SIRT7, have miR-125b binding sites
